# Parenchymal Infiltration in Primary Diffuse Leptomeningeal Gliomatosis: Dynamic Changes in Brain MRI

**DOI:** 10.3389/fonc.2017.00240

**Published:** 2017-10-09

**Authors:** Yun Jiang, Juan Chen, Jing He, Ao Pei, Jinsong Zhang, Yinhong Liu

**Affiliations:** ^1^Department of Neurology, Beijing Hospital, National Center of Gerontology, Beijing, China; ^2^Department of Radiology, Beijing Hospital, National Center of Gerontology, Beijing, China; ^3^Department of Neurosurgery, Beijing Hospital, National Center of Gerontology, Beijing, China; ^4^Department of Pathology, Beijing Hospital, National Center of Gerontology, Beijing, China

**Keywords:** primary leptomeningeal gliomatosis, parenchyma, infiltration, brain magnetic resonance imaging, dynamics

## Abstract

Primary diffuse leptomeningeal gliomatosis (PDLG) is a rare and fatal disease with no special clinical manifestations. Here, we report the dynamic brain magnetic resonance imaging (MRI) changes in a 30-year-old female PDLG patient over a 10-month period. MRI showed aggressive dilation of the subarachnoid space and the ventricular system, numerous encapsulated cysts in the subarachnoid space and the dilated cerebral sulci, diffuse reticulated or focal nodular enhancement in the subarachnoid space, as well as overall enhancement in the cystic walls. In addition to the aforementioned PDLG pathological findings, MRI also revealed non-contrasted solid lesions and a contrasted cyst-like lesion in the paraventricular areas. The dynamic and multiform neuroradiological changes help us to understand the pathological process of PDLG. Of particular interest is the discovery that parenchymal infiltration can occur in PDLG.

## Introduction

Primary diffuse leptomeningeal gliomatosis (PDLG) is associated with rapid disease progression and mortality. PDLG arises from heterotopic neuroglia tissue in the leptomeninges and spreads widely throughout the subarachnoid space (1). The majority of these neoplasms are often high grade astrocytic (2) and few are oligodendrogliomatosis (3). Compared with oligodendrogliomatosis (3, 4), the astrocyte-derived PDLG is more aggressive. Due to the non-specific and variable clinical presentation of PDLG, diagnosis relies on biopsy and histologic confirmation. As a result, most cases were only diagnosed at a late stage or postmortem. Traditionally, PDLG is confined to the leptomeninges without involvement of the brain or the spinal cord (1). Here, we report serial magnetic resonance imaging (MRI) findings from a PDLG patient over a 10-month period. In addition to dilation of the subarachnoid space and encapsulated cysts in the subarachnoid space and the dilated cortical sulci, as previously reported by other authors, the MRIs of this patient showed solid or cyst-like parenchymal infiltration. Dynamic alterations in the brain MRI findings over time help elucidate the natural history of PDLG.

## Background

**Figure 1:**
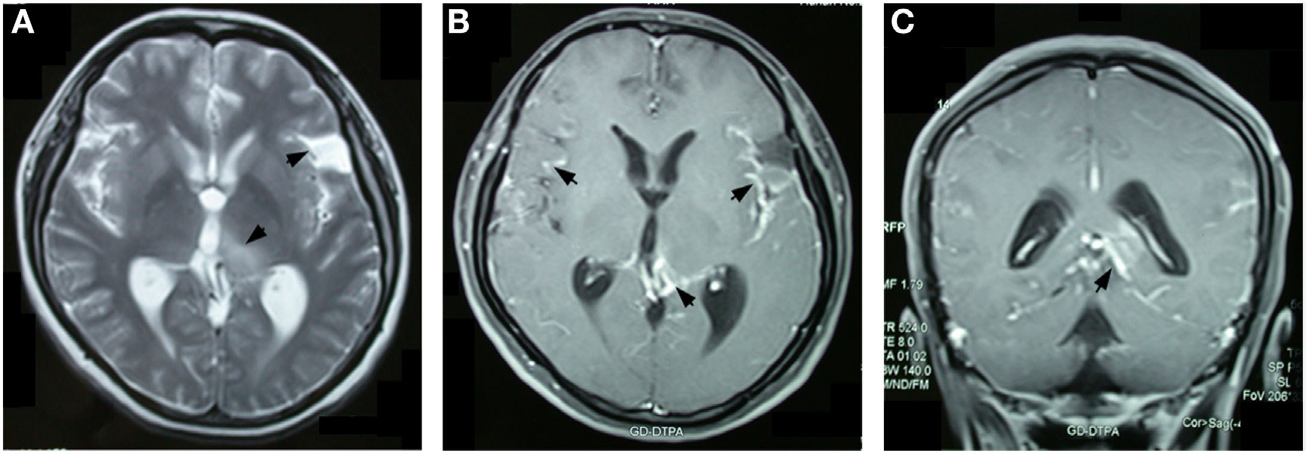
Brain magnetic resonance imaging 2 months after the onset of symptoms revealed an abnormal signal with hyper-density on T2-weighted images in the left thalamus and the dilated left Sylvian fissure **(A)**. Overall enhancement was present in the cerebral meningeal, the cerebellar tentorium, the pia mater around left the thalamus, and the quadrigeminal cistern, but not in the left thalamus **(B,C)**.

A 30-year-old, previously healthy female, was first admitted to a local hospital on June 24, 2013 with main complaints of progressive headache, nausea, vomiting, and blurred vision which had persisted for 2 months. On admission, she was afebrile and general physical examination revealed no abnormalities. Her neurological abnormalities included bilateral papilledema, vision impairment, and signs of meningeal irritation. No motor or sensory deficits were seen. Blood tests of routine, biochemistry, coagulation, thyroid function, and various immunological parameters were all in the normal range. Blood antibodies for HIV, syphilis, and tuberculosis were negative. Initial brain MRI revealed a dilated left Sylvian fissure and an abnormal signal on the medial-dorsal side of the left thalamus (Figure 1A). Diffuse enhancement was noticed in the meninges, the left thalamus adjacent ventricular ependyma, the cerebellar tentorium, and the quadrigeminal cistern, whereas no enhancement was identified in the parenchyma (Figures 1B,C). Cerebrospinal fluid (CSF) analysis in the 3-series lumbar punctures revealed opening pressures higher than 400 mmH_2_O (reference range 80–180 mmH_2_O), with protein 2.59–5.01 g/L (reference range 0.15–0.45 g/L), glucose 2.20–2.79 mmol/L (reference range 2.5–4.5 mmol/L), chloride 121.1–126.3 mmol/L (reference range 120–130 mmol/L), and WBC 3–6/mL (reference range 0–5/mL) with 80–85% of lymphocytes. Negative results were reported in the following CSF etiological investigations: polymerase chain reaction for tuberculosis, bacteria and fungi cultures, India Ink stain for Cryptococcus, and IgG and IgM antibodies targeting various viruses and parasites. CSF cytology studies did not reveal neoplasm and chest CT and abdomen CT scans did not indicate any abnormality. Tuberculous meningitis was suspected and treated with isoniazid, rifampicin, ethambutol, and pyrazinamide. Meanwhile, brain ventricular puncture and drainage were performed and this dramatically attenuated the headache symptoms. The extraventricular drainage device was removed after 2 weeks and shortly thereafter the patient suffered from generalized tonic-clonic seizures. Once the seizures were controlled, a ventricular-peritoneal shunt was placed, resulting in an improvement of the patient’s symptoms. Anti-tubercular therapy was given for 2 months, and then it was discontinued once it proved to be ineffective.

**Figure 2:**
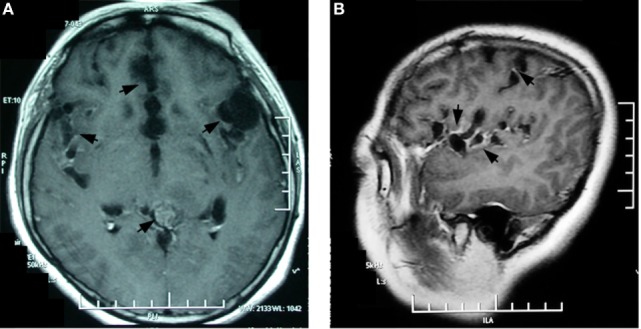
Contrasted brain magnetic resonance imaging 9 months after symptoms onset showed multiple enhanced encapsulated cyst-like lesions in the Sylvian fissure, dilated cortex sulci in the bilateral hippocampus and the medialis of bilateral frontal lobes, and a generally enhanced mass in the quadrigeminal cistern **(A)**. The sagittal image clearly shows grape-line cysts along the Sylvian fissure **(B)**.

**Figure 3:**
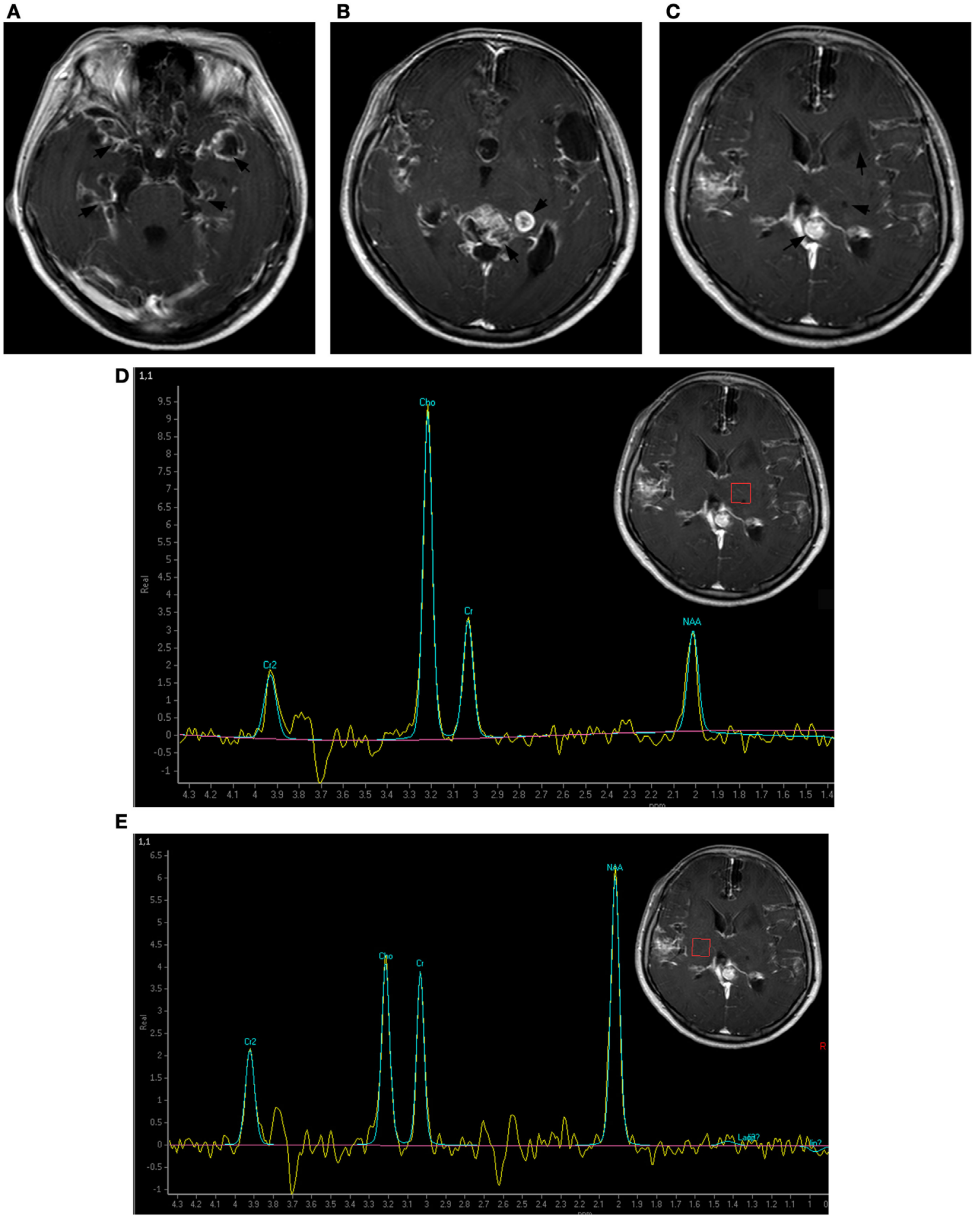
Brain magnetic resonance imaging 1 year after symptoms onset. The subarachnoid space was markedly enlarged. More contrasted cyst-like lesions were present in the Sylvian fissure, the dilated cortex, and the left basal ganglia area and a new non-contrasted solid lesion appeared in the left paraventricular white matter. The previous left thalamus lesion was consistently unenhanced **(A–C)**. Magnetic resonance spectroscopy indicated a Cho/Cr wave ratio higher than 2.5 in the left thalamus and basal ganglia area **(D)**, and approximately 1.0 in the contralateral symmetric area **(E)**.

**Figure 4:**
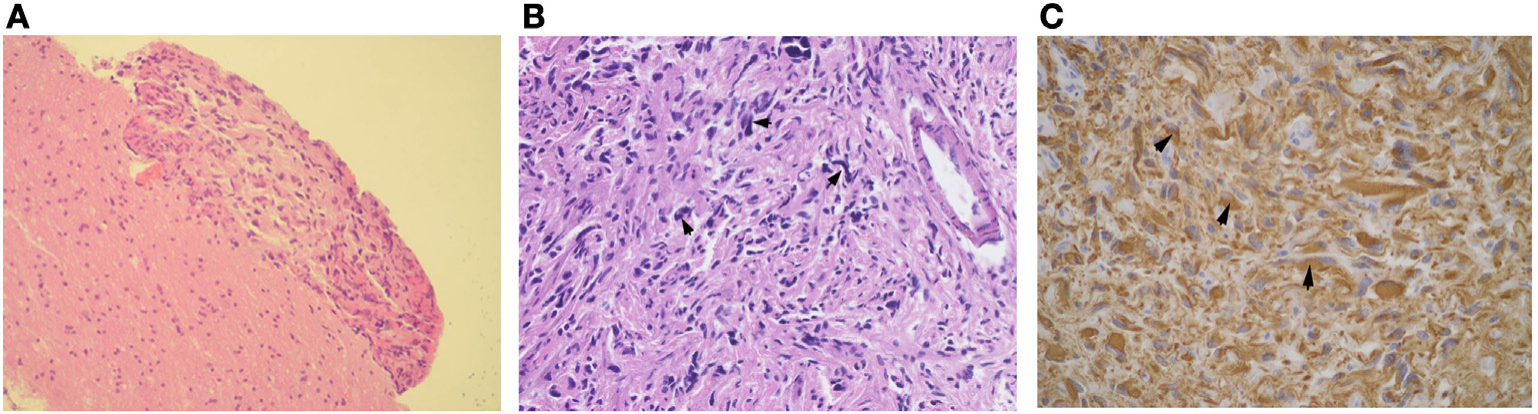
Histological examinations of a specimen obtained in left temporal lobe. Tumor tissue was localized along the cerebral meninges (HE stain, 100×) **(A)**. Spindle-shaped tumor cells with pleomorphic hyperchromatic nuclei and an increased mitotic rate spread in the subarachnoid space **(B)** (200×). The glial fibrillary acidic protein stain was strongly positive in the cytoplasm of tumor cells **(C)** (400×).

In January 2014, the patient’s severe headaches returned and her CSF pressure reached 500 mmH_2_O. Apart from a further decrease of glucose (1.88 mmol/L), her CSF analysis remained essentially unchanged. The brain MRI at this time revealed a round mass in the quadrigeminal cistern, multiple cyst signals in the Sylvian fissures, and the dilated cortical sulci in addition to the lesion in left thalamus. Overall contrast enhancement was present in the cerebral meninges, the cerebellar tentorium, the cyst walls, and the quadrigeminal cistern (Figures 2A,B). The ventricular-peritoneal shunt pressure was adjusted to 200 mmH_2_O and stereotactic biopsy of the left thalamus was performed. However, the infiltration by a few lymphocytes was the only observation. The patient’s condition deteriorated as generalized seizures became more frequent. In April 2014, she was transferred to our hospital. Upon admission, abnormal neurological findings included confusion, slow light perception, limited abduction of both eyes, grade IV of muscle strength of the right arm, general hyperactive tendon reflexes in both legs, positive Babinski’s sign on the right side, and positive meningeal signs. The third brain MRI, performed at this time, showed a non-contrasted, non-cystic lesion in the left paraventricular white matter and a contrasted cystic lesion in the left basal ganglia. All the previous changes were found to be more pronounced, including the cyst-like signals in the dilated cortical sulci, the mass in the quadrigeminal cistern, and the enlarged subarachnoid space, and general meningeal enhancement in the brain and upper segment of the cervical spinal cord (Figures 3A–C). Magnetic resonance spectroscopy (MRS) indicated a Cho/Cr wave ratio higher than 2.5 in the left thalamus and basal ganglia area (Figure 3D), and 1.0 in the right symmetric area that appeared normal on MRI (Figure 3E). Brain biopsy of the left temporal lobe was performed and spindle-shaped tumor cells were found to be spreading into the leptomeninges and subarachnoid space, which were strongly positive for glial fibrillary acidic protein (GFAP) and S100, partly positive for OLIG2 and P53, and negative for IDH1(R132H), ARTX, and NeuN (Figure 4). Ki-67 reactivity in tumor tissue was about 5%. According to 2016 World Health Organization (WHO), classification of tumors of the central nervous system, anaplastic astrocytoma, IDH-wildtype (WHO grade III) was confirmed. Combined with the extensive changes in brain MRI, PDLG was diagnosed. The patient refused treatment and was discharged from the hospital. She died 2 months later.

## Discussion

Primary diffuse leptomeningeal gliomatosis is a rare disease that likely arises from the heterotopic neuroglial tissue of the leptomeninges. Most PDLG patients die within the first year. Since 1951 (1), the diagnostic criteria of PDLG has been confined to the leptomeninges without involvement of the brain or the spinal cord. Until now, nearly all reported PDLG cases, except a few which described parenchyma infiltration (2, 5, 6), were in accordance with these criteria (7–11).

This patient underwent a series of brain MRI over a 10-month period. The main changes of her brain MRI are summarized as follows: (1) the progressive dilation of the subarachnoid space; (2) increasing cyst-like lesions in the Sylvian fissures, the dilated cortex sulci in the frontal lobes and the hippocampus; (3) a growing mass in the quadrigeminal cistern; (4) non-cyst-like lesions in the left thalamus and the left periventricular white matter; and (5) diffuse enhancement in the brain and the spinal meninges, the cyst walls, the subarachnoid space, and the mass in the quadrigeminal cistern.

In this case, the first MRI revealed a lesion in the dorsal of the left thalamus in addition to the dilation of the left Sylvian and diffuse enhancement of the meninges. Ten months later, the MRS revealed increased Cho/Cr wave ratio in the left thalamus, which is indicative of increased cellular proliferation with membrane turnover which is consistent with a neoplastic process. It is debated if the primary leptomeningeal gliomatosis infiltrated the left thalamus or if the left thalamic glioma induced secondary seeding in the leptomeninges. The solid lesion in the left thalamus was consistently non-enhanced during the 10-month period, whereas the leptomeninges around the left thalamus was strongly nodularly enhanced even in the first MRI. A fast-growing mass was present in the quadrigeminal cistern adjacent to the left thalamus. Combined with the severe dilation, as well as the overall enhancement of the subarachnoid space and multiple encapsulated cysts in the subarachnoid space and the dilated cortical sulci, it is reasonable to speculate that the solid lesion in the left thalamus is a secondary parenchymal infiltration of PDLG in the left ventricular ependyma. The parenchyma infiltration, especially the development of non-cyst lesions in the left thalamus and the periventricular white matter, challenges the traditional diagnostic criteria of PDLG.

Our serial brain MRIs depict the pathological progression of PDLG: PDLG initiates from the leptomeninges, spreads widely in the subarachnoid space and ventricular system, then extends into cortex sulci, and at last invades the periventricular parenchyma by following the cerebral perivascular space (12). In this case, during infiltration, entrapment cysts formed in the subarachnoid space, the cortex sulci, and the left basal ganglia area. Entrapment cysts in the cortex sulci were also reported earlier by Ishige (13). The placement of ventricular drainage in our patient efficiently reduced her intracranial pressure and extended her life. This made it possible to observe the terminal multiform lesions of PDLG in the brain.

Primary diffuse leptomeningeal gliomatosis can be in the form of a solitary tumor (14) or a diffuse tumor involving intracranial or spinal cord leptomeninges (8, 15). Our patient was found to have a solitary tumor in the quadrigeminal cistern and diffuse leptomeningeal gliomatosis. In 2006, Kim et al. reported a case of PDLG in which they also found a mass with intensive enhancement in the quadrigeminal cistern (9).

Primary diffuse leptomeningeal gliomatosis has no special clinical and CSF features. It presents with intracranial hypertension syndromes. The increased pressure and accumulation of the exudates in the subarachnoid space may cause paralysis of multiple cranial nerves (16). CSF tests usually reveal extremely high protein level and low glucose level. The CSF routine tests can reveal normal or slightly increased white blood cells. Even though the tumor cells spread in the subarachnoid space and ventricular system, the likelihood of finding glioma cells by CSF cytology is small (8), as shown in this patient. Repeated cytology combined with immunohistochemistry may increase the diagnostic sensitivity. The overall CSF findings are consistent with tumor, infection, and sarcoidosis. In brain MRI, the hydrocephalus and overall leptomeningeal enhancement can be seen in leptomeningeal malignancy, leptomeningeal infections, and sarcoidosis. When combined with the clinical and radiological findings, leptomeningeal involvement by an infectious process or a rapidly growing neoplasm should be highly suspected. The dynamic alterations of PDLG as described in our patient have special characteristics that could indicate PDLG. Based on brain MRI findings, a biopsy should be done to confirm the diagnosis. The early diagnosis of PDLG will enable the early treatments of radiotherapy and chemotherapy. As reported, the treatment combining radiotherapy and temozolomide could prolong the survival time (15).

## Conclusion

The dynamic and multiform neuroradiological changes help us to understand the pathological process of PDLG and highlight the possibility that parenchymal infiltration can occur in PDLG.

## Ethics Statement

The patient’s parents agreed and provided written informed consent for publication of this case report, including their child’s identifiable information.

## Author Contributions

YJ participated in the treatment of the patient, did the literature search, and drafted the manuscript. JC assisted in critical revisions of the brain MRI findings. JH participated in the treatment of the patient. AP performed the brain biopsy. JZ carefully reviewed the pathological findings. YL instructed the treatment of the patient and provided critical revisions of the manuscript for important intellectual content. All authors read and approved the final manuscript.

## Conflict of Interest Statement

The authors declare that the research was conducted in the absence of any commercial or financial relationships that could be construed as a potential conflict of interest.
